# Physiological basis of nano-silica deposition-related improvement in aluminum tolerance in pea (*Pisum sativum*)

**DOI:** 10.3389/fpls.2025.1516663

**Published:** 2025-03-11

**Authors:** Yingming Feng, Yuxin Zheng, Wei Nong, Xingyun Chen, Zeyan Wang, Peng Zeng, Xuewen Li, Shabala Sergey, Lei Shi, Min Yu

**Affiliations:** ^1^ National Key Lab of Crop Genetic Improvement, Microelement Research Centre, Huazhong Agricultural University, Wuhan, China; ^2^ International Research Center for Environmental Membrane Biology, School of Agricultural and Bioengineering, Foshan University, Foshan, China; ^3^ School of Biological Science, University of Western Australia, Crawley, WA, Australia; ^4^ Department of Educational Information Technology, Foshan University, Foshan, China

**Keywords:** pea, polyethyleneimine, nano-silica, aluminum toxicity, root tips, root border cells

## Abstract

Aluminum(Al) toxicity is a major constraint affecting crop growth in acidic soils across the globe. Excessive Al levels in such soils not only negatively affect crop growth but also have significant implications for human health. This study aimed to explore the feasibility of increasing tolerance to Al stress by creating biomineralization structures in plant roots by nano-silica, and to explore the physiological basis silicon-mediated alleviation of Al toxicity in plants. The polyethylenimine was used to induce nano-silica to form biomineralization structures on the surface of root tip and root border cells in pea (*Pisum sativum*) plants. The results showed that under Al stress conditions, the deposition of nano-silica on the cell wall of pea root border cells induced by polyethyleneimine effectively increased cell viability and reduced reactive oxygen species(ROS) production by 44%, thus slowing down the programmed cell death. Such deposition also resulted in more Al ions(Al^3+^) absorbed by the surface of the root tip, thus preventing Al^3+^ from entering the root tip and alleviating the toxic effects of Al on cell metabolism. It is concluded that polyethylenimine- induced nano-silica deposition on the cell wall endows pea root cells with Al tolerance, thus enhancing crop growth and reducing toxic Al load, contributing to food safety and human health.

## 1 Introduction

Al toxicity is a major constraint affecting crop growth in acidic soils across the globe, with a 40% of all land classified as acidic (Kochian et al., 2004). Elevated levels of Al in such soils not only negatively affect crop growth (Tabaldi et al., 2009; Liu et al., 2012) but also come with major implication for human health (Berthon, 1996; Yu et al., 2011). As the 4^th^ most abundant element in the Earth’s crust, Al primarily exists in a biologically unavailable form when soil pH is above 5. However, when soil pH drops below 4.6, aluminosilicates and aluminiferous oxides undergo weathering, resulting in the formation of Al^3+^ (Kochian et al., 2004). These ions impose numerous constraints on plant growth and development, significantly affecting agricultural productivity and ecosystem health (Rahman et al., 2024; Yan et al., 2021a, 2022). Under Al stress, plant root tips are usually the first to be affected, exhibiting symptoms such as root stubbiness, necrotic shedding of epidermal cells at the root tip, arrest in the root tip expansion, overproduction of ROS, and reduction in the number of lateral roots (Li et al., 2018; Feng et al., 2019; Yang et al., 2022; Tao et al., 2023). Many root cells, including those in apical meristem and root border cells (RBCs) undergo programmed cell death (PCD) when exposed to Al (Wu et al., 2018; Jalili et al., 2022; Huang et al., 2022).

It has been demonstrated that Al load in plants can be partially reduced by silicon, thereby mitigating the effects of Al toxicity (Doniak et al., 2014; Feng et al., 2019; Xiao et al., 2022; Nabipour et al., 2022). Polyethyleneimine (PEI) is a positively charged polyelectrolyte that can induce the deposition of nano-silica on the surface of plant cells thus alleviating the toxic effect of Al^3+^ on cells. In the past, PEI-induced nano-silica deposition has been successfully used to reduce Al toxicity in yeast and plant suspension cells (Lee et al., 2014; He et al., 2015). But whether this method can be effectively applied to higher plant cells or roots remains unclear.

The root border cells (RBC), as one of the structural and functional components of the root system, are easy to detach from the root tip and could survive for a longer period in autonomous metabolism, making them an ideal model cell for studying cell physiological functions under heavy metal stress (Feng et al., 2023). Therefore, in this study, we employed pea root tips and RBCs as the model systems to explore the feasibility of using the polyethyleneimine template technology to induce the biomineralization deposition of nano-silica on their surfaces. We also explored the physiological mechanism by which PEI-induced nano-silica deposition mitigates Al toxicity in plants through the determination of physiological changes in the root tips and RBC. This work provides a theoretical basis for developing bionanotechnological approaches to alleviate of Al toxicity in crops grown under acidic soil conditions.

## 2 Materials and methods

### 2.1 Test material

The pea seeds of the test material were Chinese pea (*Pisum sativum* cv ZW6). The details regarding seed selection and crop cultivation are provided in our previous work (Feng et al., 2019). When the primary root tip reached approximately ∼2 cm in length, it was utilized for the collection of RBCs as described elsewhere (Feng et al., 2019). The roots were then transferred to the hydroponic container containing 1/4 Hogland nutrient solution for various experiments, as described below. The experiment was conducted under controlled temperature conditions, with temperatures maintained at 26 ± 2°C and 11h photoperiod.

### 2.2 Test methods

#### 2.2.1 Nano-silica biomineralization deposition in root border cells and Al stress treatment

Nano-silica biomineralization deposition was performed according to the method established by Lee et al. (2014), with some modifications. The collected RBCs solution was centrifuged, and the supernatant was collected and treated with 0.005 g/L PEI (Polyethyleneimine, Sigma) at pH 7.0 containing 50 mmol/L HEPES buffer for 10 min. The mixture was then centrifuged and the supernatant aspirated, washed twice with 50 mmol/L HEPES (pH 7.0) buffer, and then treated with 1 mmol/L Si solution (methyl orthosilicate, Macklin) containing 50 mmol/L HEPES buffer for 20 min. The mix was centrifuged and aspirated once more, washed twice with 50 mmol/L HEPES (pH 7.0) buffer, and then stored for further use. Al stress treatment was performed by treating the nano-silicon induced deposited cells with 100 µmol/L AlCl_3_ (made in 5 mM Homo-PIPES buffer, pH 4.5) for 1h. The same solution (minus Al) was used as a control. Altogether, there were four treatments in the experiment, with two levels of Al and two levels of Si (e.g., -Si-Al; -Si+Al; +Si-Al; and +Si+Al).

#### 2.2.2 Nano-silica deposition in root tips and Al stress treatment

After cultivating the planted pea seedlings for 2d, the root tips of the main roots were cut about 3 mm and treated with 0.03 g/L PEI solution (pH 6.0, containing 0.5 mmol/L CaCl_2_ solution) for 4h. Excised tips were then treated with 10 mmol/L Si solution (pH 6.0, containing 0.5 mmol/L CaCl_2_ solution) for 8h and left acclimating in 0.5 mmol/L CaCl_2_ solution (pH 4.5) for 12h. 100 μmol/L AlCl_3_ (pH 4.5) was then added to the solution for 24h, while control roots we kept in 0.5 mmol/L CaCl_2_ solution (pH 4.5). Overall, there were four treatments in the experiment (as in 2.2.1).

#### 2.2.3 Root border cells viability assay

Trypan Blue (TB) staining was used according to Feng et al. (2019) for cell viability assays. Root border cells were exposed to 100 μmol/L AlCl_3_ (with 1 mM CaCl_2_, pH 4.5) for 1 h. Cell viability was then examined under a microscope by Trypan Blue (0.5%) exclusion assay.

#### 2.2.4 Root border cell reactive oxygen species assay

ROS were detected according to the method of Xia et al. (2022) with modifications. 50μg CM-H_2_DCFDA was dissolved in 86.5μL of anhydrous ethanol in a tube to prepare a 1 mmol/L master mix. Subsequently, it was diluted to a final concentration of 20 µmol/L using a 1 mmol/L CaCl_2_ solution. For treatment, equal volumes of this dye solution and border cell suspension were mixed, and cells were incubated for 30 min in the dark, shaking them every 5 min to ensure uniform staining. After staining the cells were washed twice with 1 mmol/L CaCl_2_ solution. Stained cells were when observed and the intensity of the fluorescent signal was measured using a laser scanning confocal microscope (LSCM; ZEISS LSM 980; Oberkochen, Germany), under an excitation wavelength of 488 nm and an emission wavelength of 510 nm-530 nm. Enhanced fluorescence intensity indicated increased ROS content.

#### 2.2.5 Localization of active Al in root border cells

After Al treatment, 100μL of cell suspension was mixed with the same volume of Morin fluorescent stain at a concentration of 0.01%. And incubated for 20 min in the dark. Homogeneous staining was achieved by shaking every 5min using a micro vortex mixer (XW-80A; Shanghai, China). After staining, cells were centrifuged in a high-speed centrifuge (Eppendorf, Centrifuge 5425, Germany) for 2 min at a speed of 8000 rpm, the supernatant was discarded and the precipitate washed with 50 mmol/L HEPES buffer at least three times, then stored in the buffer. Cells were observed and the intensity of the fluorescent signal was measured using CLSM with an excitation wavelength of 405 nm and an emission wavelength of 500 nm-530 nm. The relative fluorescence intensity correlated with the amount of active Al. 20 cells were measured for each group.

#### 2.2.6 Lateral root growth assay

Roots were scanned with a root scanner (Epson Expression 11000 XL, Beijing, China) immediately before treatments, and then exposed to a combination of Al and Si deposits. The scan was then repeated. The length of lateral roots was measured at the same position of each seedling before and after Al treatment by Image J software, and the following calculations were made:

Lateral root increment (mm) = L_a_-L_b_, where L_a_ is the length (mm) after the treatment, and L_b_ is the length before the treatment;
Root relative elongation rates (RRE) = Z_2_/Z_1_*100%, where Z_1_ is the length increment for -Si-Al treatment, and Z_2_ is the root length increment for other treatment groups.

#### 2.2.7 ROS accumulation in root tips

Twenty lateral roots were stained with CM-H_2_DCFDA fluorescent staining solution at a concentration of 10 μmol/L for 25 min in the dark and then washed three times with distilled water. Segments were observed and photographed under a stereo fluorescence microscope (Olympus SZX16; Tokyo, Japan), and the fluorescence intensity was measured, to determine the extent of ROS accumulation in the root tips. Three independent experiments were conducted, with 10 lateral roots in each group.

#### 2.2.8 Root tip callose content assay

Root segments were collected as described in 2.2.7. Segments were treated with FAA fixative (containing 10% formaldehyde, 5% glacial acetic acid, 45% ethanol, pH 8.5) for 24 h and then washed three times with distilled water. Roots were then softened with phosphate buffer (PB, pH 8.5) for 30 min, washed three times, and then treated with 0.01% aniline blue for 30 min, followed by another wash. The root tips were imaged using a stereo fluorescence microscope (Olympus SZX 16; Tokyo, Japan), and the intensity of blue fluorescence in various root zones was quantitatively assessed using ImageJ software. The relative blue fluorescence intensity correlated with the content of callose. Three independent experiments were conducted, with 10 lateral roots in each group.

#### 2.2.9 Root tip hematoxylin staining

Root segments were collected as described in 2.2.7, placed in a 1.5 mL centrifuge tube, and stained with Hematoxylin staining solution at a concentration of 0.1% for 10 min. Roots were washed three times with distilled water and then imaged using a stereo fluorescence microscope (Olympus SZX 16; Tokyo, Japan). The complexation of hematoxylin and Al^3+^ results in a purple coloration. The darker color intensity corresponds to increased Al content. Three independent experiments were conducted, with 10 lateral roots in each group.

#### 2.2.10 Localization of active Al in root tips

After Al treatment, root segments were collected as described in 2.2.7. Segments were placed in a 1.5 mL centrifuge tube and stained with a 0.1% concentration of Hematoxylin staining solution for 20 min in the dark. The roots were subsequently washed three times with distilled water and then imaged using a stereo fluorescence microscope (Olympus SZX 16; Tokyo, Japan). The intensity of green fluorescence in various root zones was quantitatively assessed using ImageJ software. The relative green fluorescence intensity correlated with the amount of active Al. Three independent experiments were conducted, with 10 lateral roots in each group.

## 3 Results and analysis

### 3.1 Effect of nano-silica deposition on the survival of pea RBCs under Al stress

**Figure 1:**
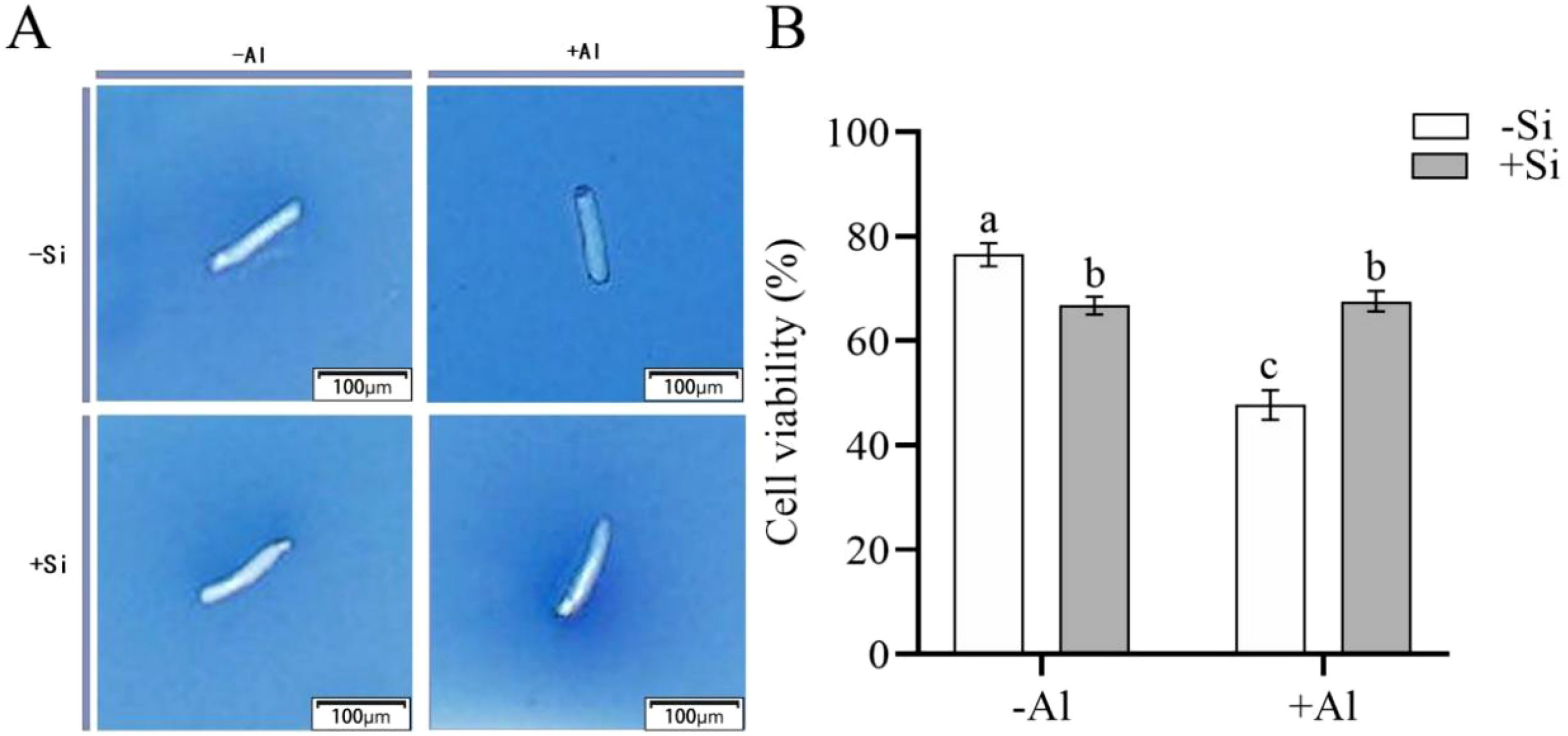
Effect of PEI-induced deposition of nano-silica and Al on the viability of pea root border cells. **(A)** Pictures after trypan blue staining of RBCs after different treatments; **(B)** Comparison of survival rate of RBCs after different treatments. Values are mean ± SD (n = 60; 20 cells measured in three independent sets of experiments). Different lowercase letters indicate a significant difference at P<0.05.

The viable cells have semipermeable membranes and are not permeable to trypan blue (TB) so have a white color. When the integrity of the cellular membrane is compromised, TB enters the cells to color them blue. As shown in **Figure 1**, in the absence of Si cells viability was significantly reduced by Al, dropping from 76.5% for -Si-Al group to 47.6% for -Si+Al treatment. However, the viability of RBCs treated with polyethylenimine-coated silicon (PEI-Si) was not significantly affected by Al as compared to appropriate control (+Si+Al vs +Si-Al treatments). The results indicate that Al stress results in a significant loss of cell viability, while SiO_2_ deposition on the cell surface via PEI can effectively increase cell survival and reduce the mortality of RBCs.

### 3.2 Effect of Al stress on ROS levels in pea RBCs and their modification by nano-silica deposition

**Figure 2:**
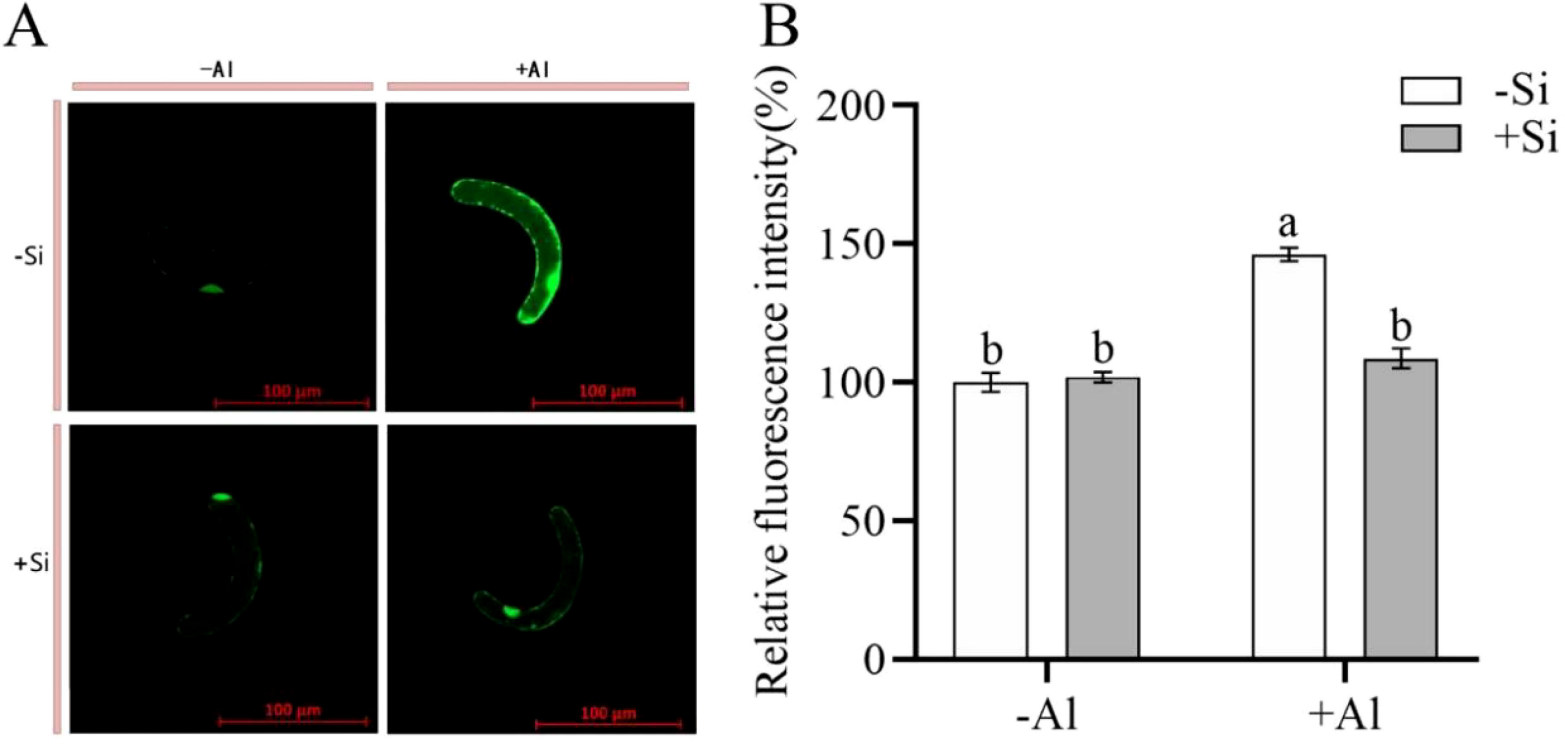
Effect of PEI-induced deposition of nano-silica and Al on ROS content in RBC. border cells. **(A)** Fluorescence staining of reactive oxygen species in pea RBCs; **(B)** A relative fluorescence intensity of pea root border cells after CM-H_2_DCFDA fluorescent staining was calculated. Values are mean ± SD (n = 60; 20 cells measured in three independent sets of experiments). Different lowercase letters indicate a significant difference at P<0.05.

When plants are stressed, they accumulate large amounts of ROS in their tissues. CM-H_2_DCFDA is a fluorescent dye that is commonly utilized for detecting for detecting ROS in plant cells. When observed under a confocal laser microscope, a brighter color (higher fluorescence intensity) signaling increased ROS production. As shown in **Figures 2A, B**, cells under Al-free conditions (-Si-Al) showed a weak fluorescence signal, whereas cells exposed to Al (-Si+Al) the ROS production is increased (by 46%). This finding indicates that Al stress can induce ROS production in pea RBCs, prompting the burst of reactive oxygen species and triggering the programmed cell death (PCD) of RBCs, whereas nano-silica deposition on the cell surface can effectively inhibit the ROS burst, thereby preventing PCD.

### 3.3 Effect of Al stress on active Al localization in pea RBCs by nano-silica deposition

**Figure 3:**
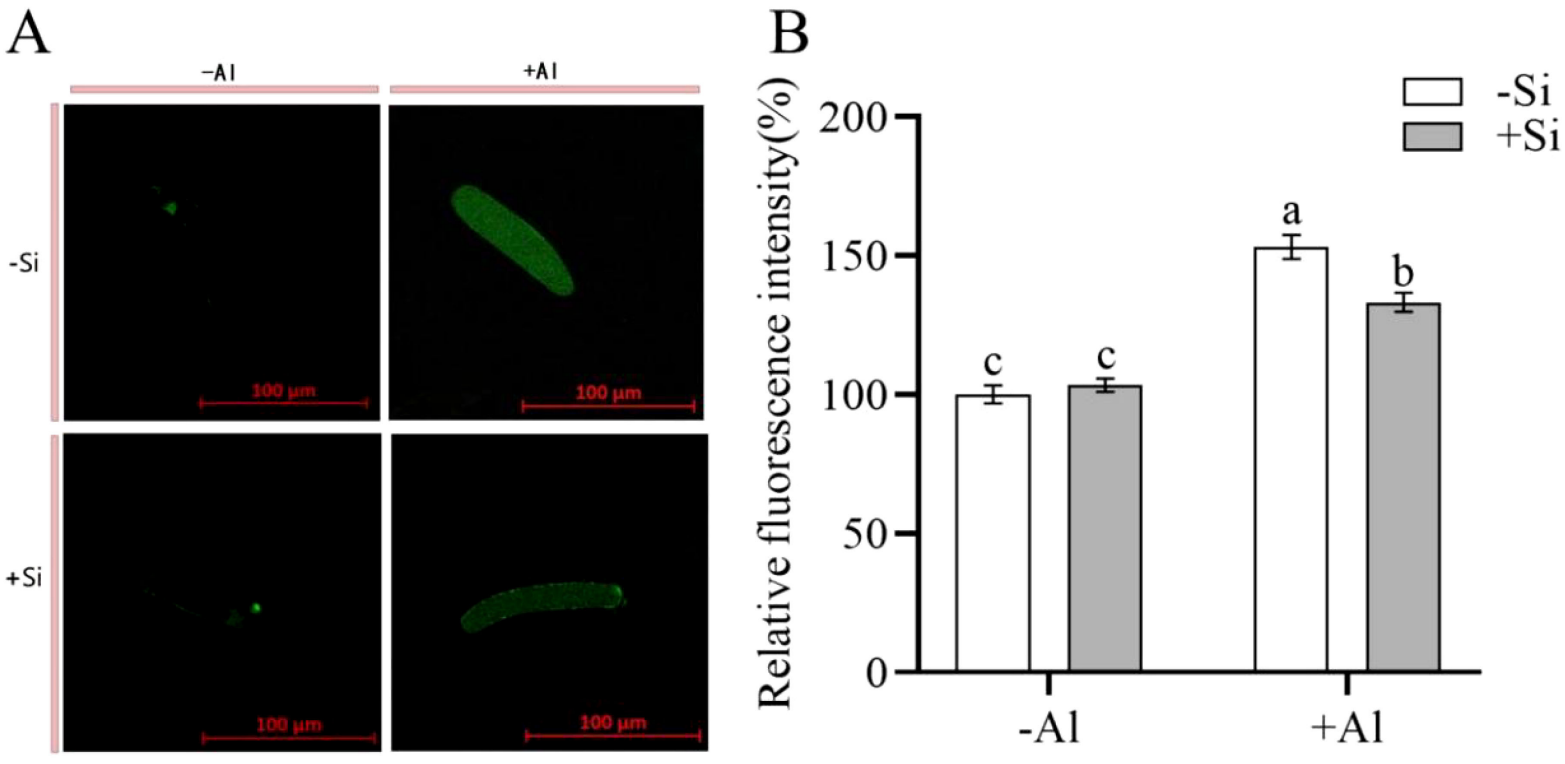
Effect of PEI on the localization of active Al in pea root border cells cultured with nano-silica and Al. **(A)** images of morin-stained pea root border cells; **(B)** A relative fluorescence intensity of pea root border cells after Morin staining was calculated. Values are mean ± SD (n = 60; 20 cells measured in three independent sets of experiments). Different lowercase letters indicate a significant difference at P<0.05.

Morin serves as a fluorescent probe that is commonly utilized for the detection of cellular active Al localization. Upon combining with unstable Al3+ within the cell, it forms a stable green fluorescent complex, Morin-Al, wherein the intensity of the fluorescent signal is proportional to the quantity of active Al present in the cell. As depicted in **Figures 3A, B**, the fluorescence of the cells under Al-free condition (-Si-Al) was extremely weak, while the fluorescence intensity of the cells under Al treatment (-Si+Al) was significantly increased by 53.03%. Under Al stress, the green fluorescence intensity of RBCs (+Si+Al) with PEI-induced nano-silica deposition was relatively weaker, with a significant decrease of 19.92% compared with uninduced root margin cells (-Si+Al). The experimental results showed that PEI-induced nano-silica caused Al to be deposited on the protective nano-silica layer, which reduced the binding of Al to the cells and significantly reduced the accumulation of active Al in the cells, thereby protecting the cells from damage.

### 3.4 Effects of nano-silica deposition on lateral root growth under Al stress

**Figure 4:**
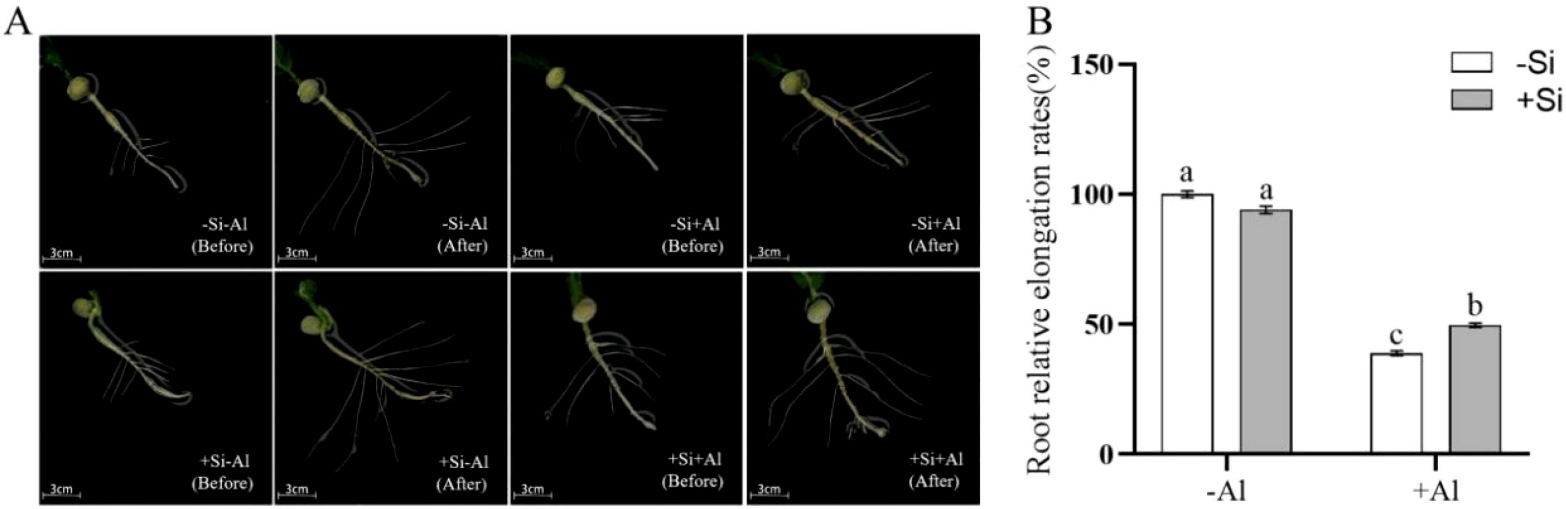
Effects of PEI-induced nano-silicon deposition on the growth of pea lateral roots under Al stress. **(A)** The growth of lateral roots; **(B)** Growth rate of lateral root. Values are mean ± SD (n = 50). Different lowercase letters indicate a significant difference at P<0.05.

Root elongation is significantly inhibited when the plant root tip is exposed to Al toxicity, as Al accumulates mainly in the cell wall in this region and is first in contact with Al^3+^. Under silica-free conditions, Al exposure reduced pea root elongation and lateral root growth by ∼ 50% (**Figure 4**) as compared to -Al treatment. PEI-induced nano-silica deposition on the surface of root tips mitigated the above reduction in both root length increment (**Figure 4A**) and the relative growth rate (**Figure 4B**).

### 3.5 Effects of Al stress and nano-silica deposition on ROS accumulation in roots

**Figure 5:**
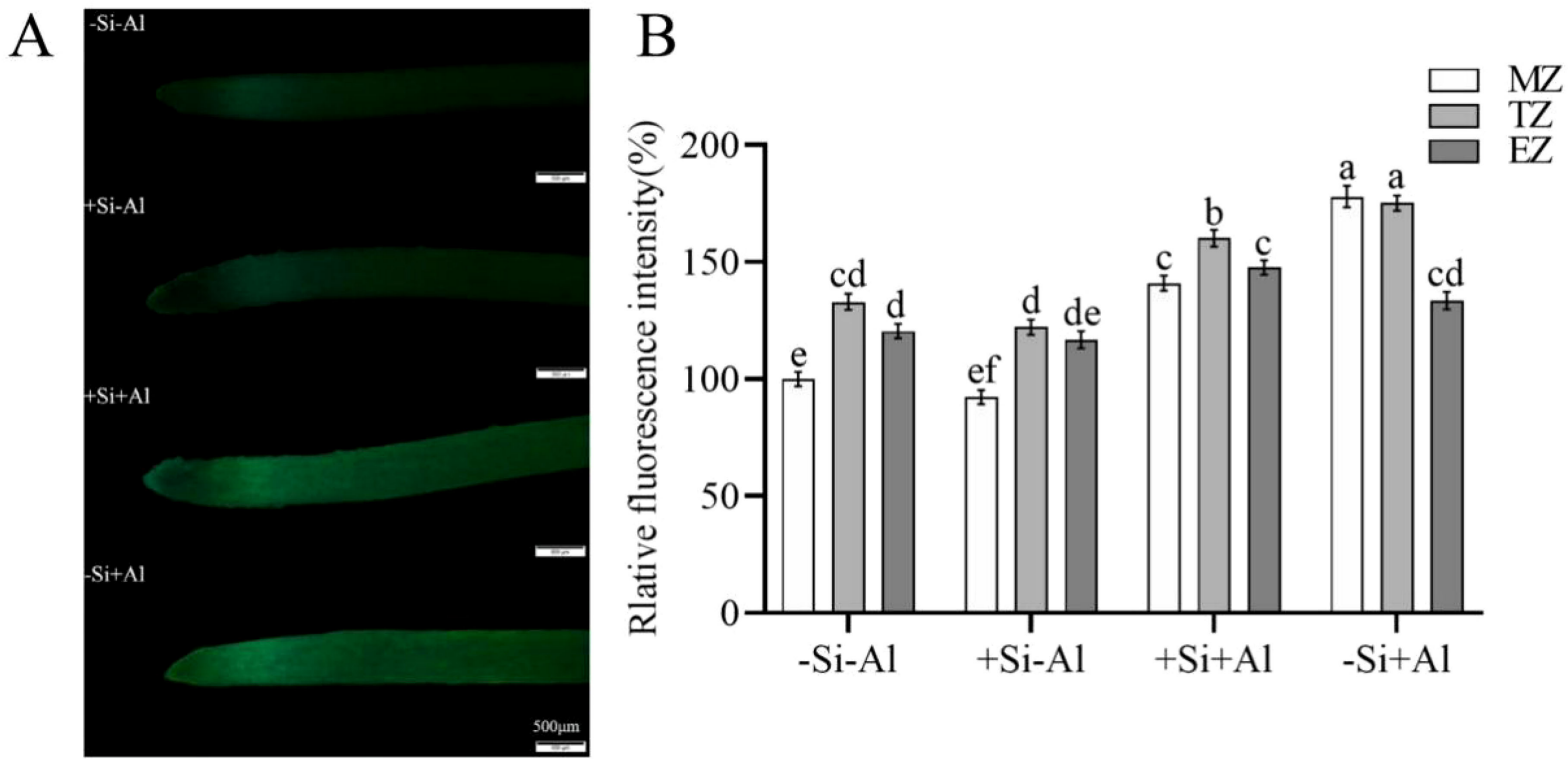
Effects of PEI- induced nano-silica deposition on ROS content in pea root tips under Al stress. **(A)** Fluorescence ROS staining in root tips; **(B)** A relative fluorescence intensity of root tips’ meristematic zone (MZ), transition zone (TZ) and elongation zone (EZ) after CM-H_2_DCFDA staining was calculated. Values are mean ± SD (n = 20). Different lowercase letters indicate a significant difference at P<0.05.

There was no significant difference in the ROS content in pea root tips under -Si and +Si conditions in the absence of Al^3+^ (**Figure 5A**). The overall fluorescence of the root tips was rather weak, with the strongest signal emanating from the transition zone of the root tips (with high metabolic activity). Al exposure has boosted ROS production, and in the absence of Si, there was a significant difference in ROS in pea root tips under -Al and +Al conditions in each zone (**Figure 5B**). Si deposition reduced ROS production in root meristem (MZ) and transition zone (TZ) but not elongation zone (EZ)(**Figure 5B**). Thus, PEI-induced nano-silica deposition may reduce the burst of ROS resulting from Al exposure and reduce the damage to root tips.

### 3.6 Effect of Al stress and nano-silica deposition on the callose content in pea root tips

**Figure 6:**
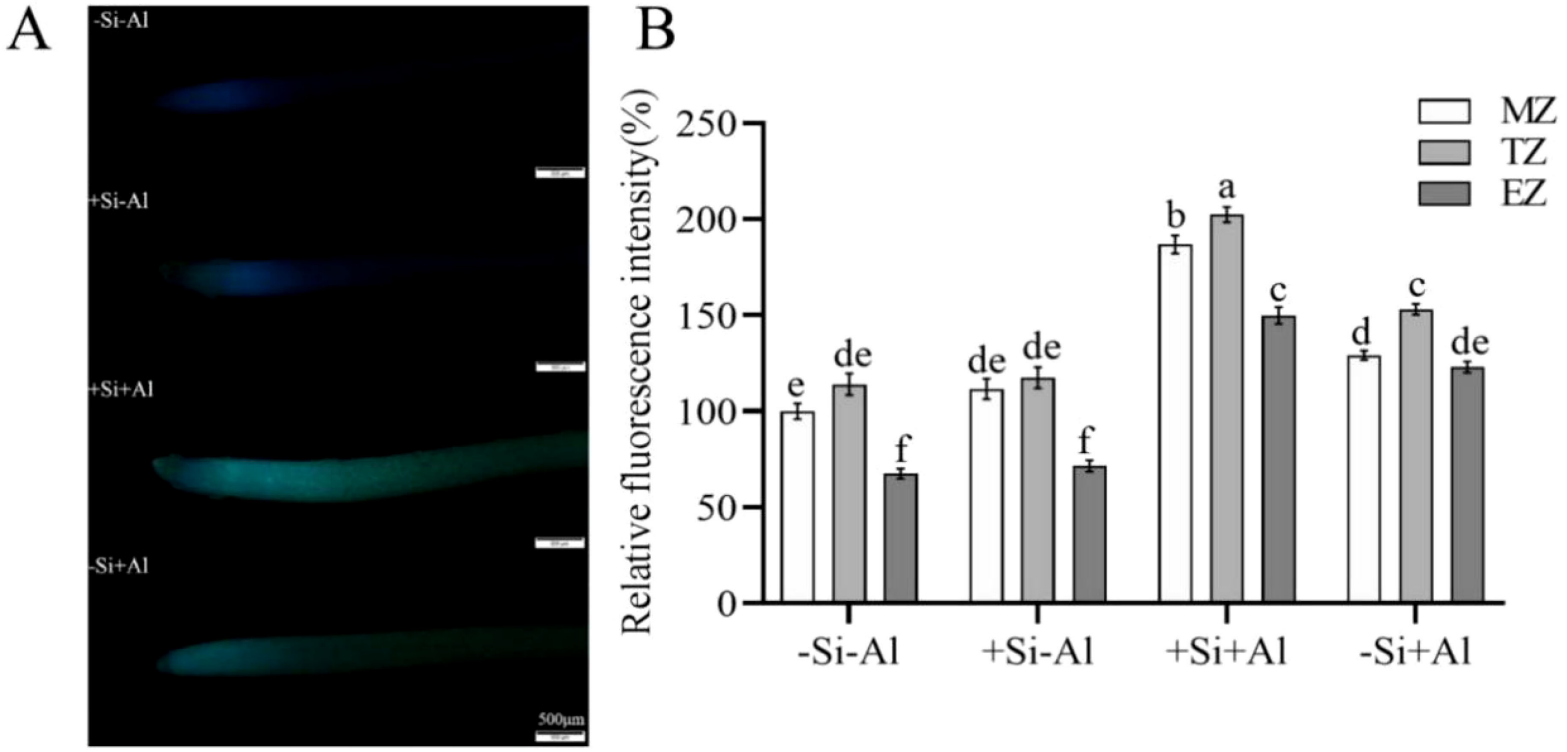
Effect of PEI-induced nano-silica deposition on callose content in root tip of pea under Al stress. **(A)** Fluorescence staining of callose in pea root tips; **(B)** A relative fluorescence intensity of root tips’ meristematic zone (MZ), transition zone (TZ) and elongation zone (EZ) after Morin staining was calculated. Data are mean ± SD (n = 20). Different lowercase letters indicate a significant difference at P<0.05.

Callose synthesis is an immediate stress response of plant root tip under Al stress and is considered one of the factors inhibiting the growth of the root tip. There was a positive correlation between the amount of Al uptake in the root tip and the amount of callose formation (stronger fluorescent signal; **Figure 6A**). No significant difference in the callose content was observed between -Si and +Si conditions in the Al-free treatment. As expected, the lowest signal originated from the rapidly expanding elongation zone (EZ). The intensity of the fluorescent signal increased by 44% after Al exposure in -Si treatment (**Figure 6B**; a mean value of signals from meristematic, transition, and elongation zones). The physiological rationale beyond this observation may be that PEI-induced nano-silica adsorbed more Al to accumulate on the protective layer of nano-silica under Al stress, which increased the adsorption sites for Al and thus prevented Al from entering the root.

### 3.7 Effect of Al stress and nano-silica deposition on hematoxylin staining of pea root tips

**Figure 7:**
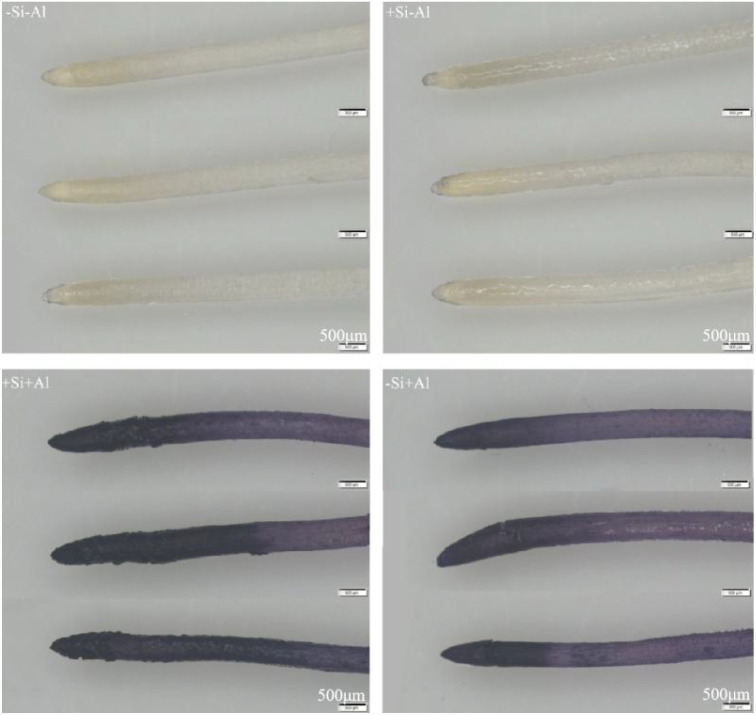
Effects of PEI-induced nano-silica deposition on hematoxylin staining of pea root tip under Al stress.

The Al content and the distribution patterns on the surface of pea root tips were observed using hematoxylin staining, with darker colors indicating higher Al accumulation. In the absence of silicon, the Al content on the surface of the root tips was significantly increased upon Al exposure (**Figure 7**). The strongest deposition (darker colors) was in the meristematic zone and transition zone, and the tips appeared swollen and ruptured. Under Al conditions, the surface Al content of pea root tips with +Si was significantly increased in the meristematic zone and transition zone than that of -Si, Notably, the surface of root tips treated with +Si appeared uneven, while the surface of elongation zone exhibited lighter colors, potentially due to the epidermal cells in this region having died and fallen off under high concentrations of Al stress, exposing the inner cells with minimal coloration. These observations suggest that the deposition of nano-silica caused more Al to be deposited on the surface of the root tip in the meristematic and transition zones, which led to an increase in the Al content of the root section, and the Al was deposited on the protective layer of nano-silica after the PEI-Si treatment to alleviate the effect of Al toxicity.

### 3.8 Effects of Al stress and nano-silica deposition on the localization of active Al in pea root tips

**Figure 8:**
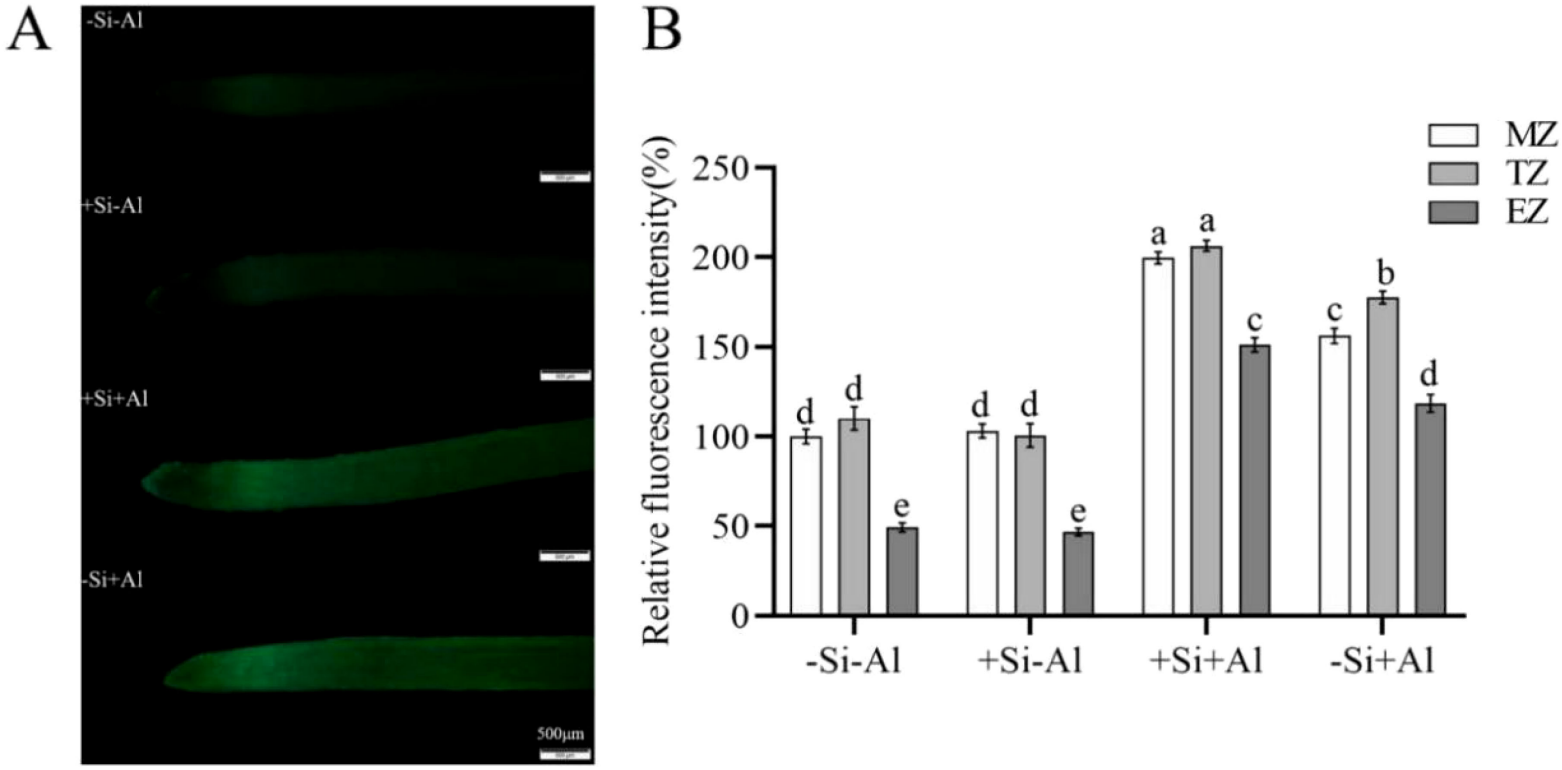
Effect of PEI- induced nano-silica deposition on active Al localization in pea root tips under Al stress. **(A)** Fluorescence staining diagram of active Al in root tips; **(B)** A relative fluorescence intensity of root tips’ meristematic zone (MZ), transition zone (TZ) and elongation zone (EZ) after Morin staining was calculated. Data are mean ± SD (n = 20). Different lowercase letters indicate a significant difference at P<0.05.

Morin is a fluorescent probe used to determine the accumulation of active Al in the cell, specifically manifested as green fluorescence, with the fluorescence intensity being proportional to the content of active Al in the cell. Under control conditions (no Al stress), the fluorescent morin signal was rather weak and not different between +Si and -Si treatments (**Figure 8**). The strongest signal originated from the transition zone. The addition of Al increased morin fluorescent signal, showed obvious peaks in the meristematic and transition zones, and the relative fluorescence value of -Si+Al was 74% higher than that of -Si-Al (**Figure 8B**; the average values of meristematic, transition, and elongation zones). The overall fluorescence of the root tips of +Si+Al was ∼ 40% stronger compared with -Si+Al (**Figure 8B**; the mean values of the meristematic, transition, and elongation zones). Thus, the PEI-induced deposition of nano-silica led to the deposition of Al^3+^ on the protective layer of nano-silica, which formed more Al^3+^ attachment points to prevent the entry of Al^3+^ into the root tips, protecting them from Al toxicity.

## 4 Discussion

### 4.1 Physiological mechanisms by which cell-level nano-silica deposition confers Al resistance

Al toxicity hinders plant growth and photosynthesis, affecting cell integrity and accelerating metabolic imbalance, which stunts the growth of plants and reduces their quality and yield (Tyagi et al., 2020; Yan et al., 2023). Silicon is ubiquitous and plays a beneficial role in plant biology, favoring plant growth, and its presence in the intracellular or extracellular compartments of plants contributes to improving the mechanical strength of cells and mitigating biotic and abiotic stresses (Zhang et al., 2015; Pang et al., 2023). Biomineralized SiO_2_ particles at the nanoscale have their unique features superior to those of SiO_2_ particles, and these nanoparticles can enter and interact with cells (Feng et al., 2022).

PEI is a positively charged polyelectrolyte capable of inducing the deposition of nano-silica on the surface of plant cells, which has been successfully applied to yeast cells and rice suspension cells. These studies showed that the induced nano-silica deposition can inhibit cadmium ions from entering into the cells, effectively alleviating their toxic effects (Lee et al., 2014; He et al., 2015; Ma et al., 2015, 2016; Feng et al., 2023). Programmed cell death (PCD) is an inherent mechanism of cell death that is controlled by specific genes and protein pathways. PCD has gained significant attention in the fields of plant physiology and molecular biology in recent years. Aluminum stress induces the accumulation of excessive ROS in plant chloroplasts, mitochondria, and peroxisomes (Delisle et al., 2001; Yamamoto et al., 2003; Shavrukov and Hirai, 2015), resulting in PCD (Achary and Panda, 2009). High ROS concentrations can change the antioxidant status of cells, leading to spontaneous cell death (Ye et al., 2021). Research has shown that environmental stresses disturb the equilibrium between the creation and removal of ROS, resulting in increased cellular ROS levels (Farooq et al., 2019). The accumulation of cellular ROS acts as a signaling molecule to modulate gene expression or triggers oxidative damage to proteins, DNA, and lipids, leading to cell damage and even cell death (Farooq et al., 2019; Jiang et al., 2021). Our study showed that the deposition of nano-silica on RBCs can mitigate the release of ROS triggered by aluminum stress (**Figure 2**) and decrease the content of active aluminum in RBCs (**Figure 3**), thus improving the viability of RBCs when exposed to aluminum stress (**Figure 1**).

### 4.2 Physiological mechanisms of Al resistance conferred by nano-silica deposition at the root tip

The root cap is an important factor affecting the inter-root microbial community (Tariq et al., 2024). As specialized cell populations aggregated around the root cap, RBCs exhibit robust protection against Al stress through the deposition of nano-silica induced by PEI. Furthermore, the root tip represents the most sensitive region in plants to Al toxicity in plants (Yan et al., 2021b; Zhu et al., 2022; Jiang et al., 2023). This explains our choice of selecting these tissues to explore the possibility of alleviating Al toxicity by inducing nano-silica by PEI.

Under Al stress, root tip elongation and growth were inhibited, resulting in a decrease in the number of lateral roots and a great limitation of root elongation; these inhibitory effects were alleviated by PEI-induced nano-silica deposition (**Figure 4**). The alleviation observed may be attributed to conditions of cell viability under Al exposure conditions.

## 5 Conclusion

**Figure 9:**
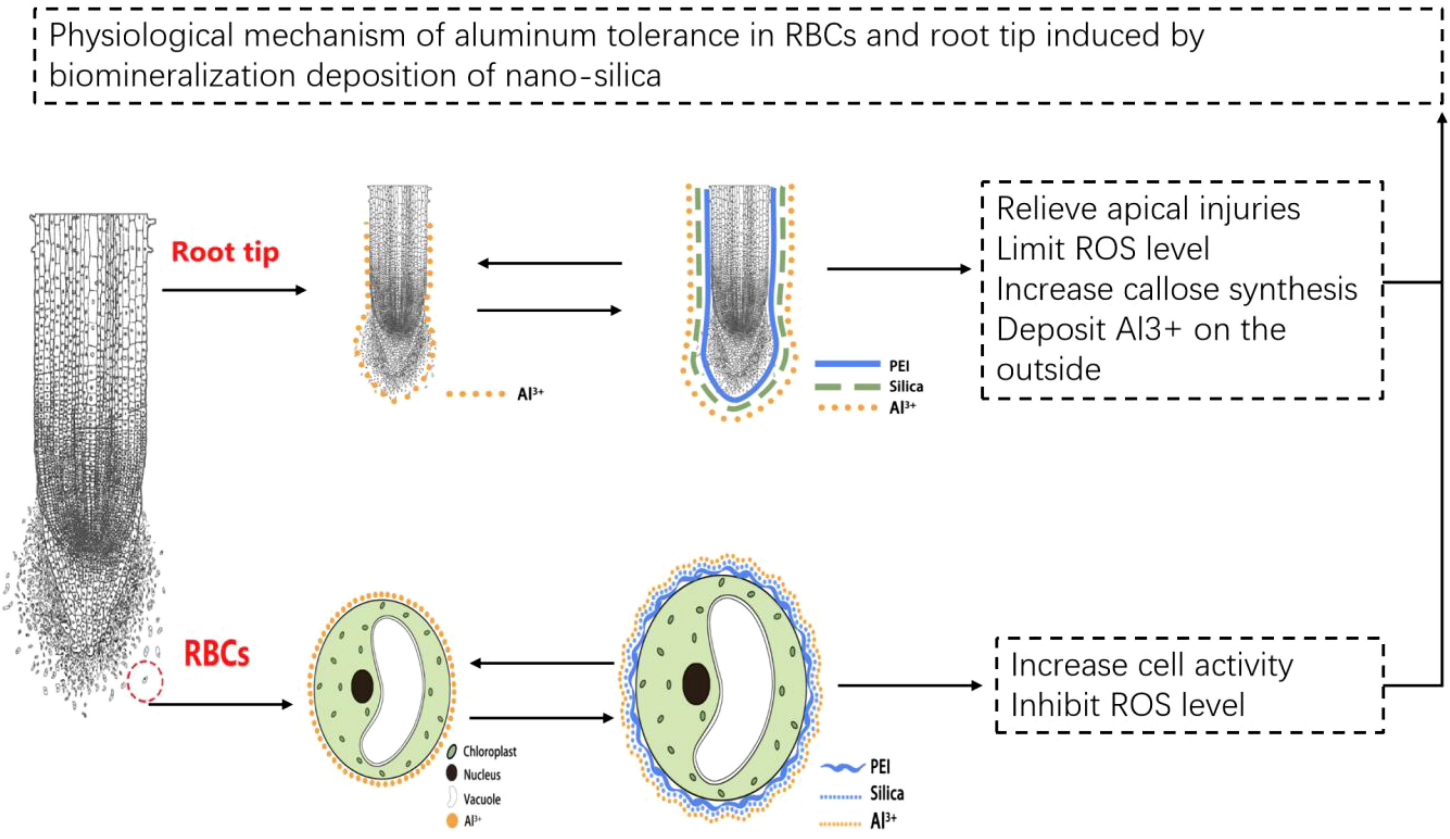
Tentative model of Al tolerance in RBCs and root tip induced by biomineralization deposition of nano-silica.

PEI-induced deposition of nano-silica on pea RBCs and root tips mitigated the toxic effects of Al stress and improved plant tolerance to Al stress. The mechanistic basis of this process can be summarized as follows (**Figure 9**). PEI-induced nano-silica deposition on RBCs slowed down programmed cell death and enhanced cell survival, due to mitigating stress-induced increase in ROS content. Silica nanoshells also formed a protective layer on the cell wall of the pea root tip, which adsorbed more Al and deposited it on the root surface and prevented it from entering into the tip. In this study, nano-silica was artificially coated on the surface of plant cells, thereby establishing the research foundation for the technology of forming silica nanoshells through biomineralization. Additionally, it also provides a new idea for the research of improving plant resistance. This technology can be applied to crops growing in acidic soil and may increase crop production.

## Data Availability

The original contributions presented in the study are included in the article/supplementary material. Further inquiries can be directed to the corresponding authors.

## References

[B1] Achary V. M. M. Panda B. B. (2009). Aluminum-induced DNA damage and adaptive response to genotoxic stress in plant cells are mediated through reactive oxygen intermediates. Mutagenesis 25, 201–209. doi: 10.1093/mutage/gep063 19955331

[B2] Berthon G. (1996). Chemical speciation studies in relation to aluminium metabolism and toxicity. Coordination Chem. Rev. 149, 241–280. doi: 10.1016/S0010-8545(96)90030-2

[B3] Delisle G. Champoux M. Houde M. (2001). Characterization of oxalate oxidase and cell death in Al-sensitive and tolerant wheat roots. Plant Cell Physiol. 42, 324–333. doi: 10.1093/pcp/pce041 11266584

[B4] Doniak M. Barciszewska M. Z. Kazmierczakk J. Kazmierczak A. (2014). The crucial elements of the ‘last step’ of programmed cell death induced by kinetin in root cortex of V. faba ssp. minor seedlings. Plant Cell Rep. 33, 2063–2076. doi: 10.1007/s00299-014-1681-9 25213134

[B5] Farooq M. A. Niazi A. K. Akhtar J. Saifullah N. Farooq M. Souri Z.. (2019). Acquiring control: The evolution of ROS-Induced oxidative stress and redox signaling pathways in plant stress responses. Plant Physiol. Biochem. 141, 353–369. doi: 10.1016/j.plaphy.2019.04.039 31207496

[B6] Feng Y. Han H. Nong W. Tang J. Chen X. Li X.. (2023). The biomineralization of silica induced stress tolerance in plants: a case study for aluminum toxicity. Plant Signaling And Behavior 18, 1559–2324. doi: 10.1080/15592324.2023.2233179 PMC10337488 37431740

[B7] Feng Y. Kreslavski V. D. Shmarev A. N. Ivanov A. A. Zharmukhamedov S. K. Kosobryukhov A.. (2022). Effects of iron oxide nanoparticles (Fe_3_O_4_) on growth, photosynthesis, antioxidant activity and distribution of mineral elements in wheat (*Triticum aestivum*) plants. Plants-Basel 11, 1894. doi: 10.3390/plants11141894 35890527 PMC9322615

[B8] Feng Y. Li X. Guo S. Chen X. Chen T. He Y.. (2019). Extracellular silica nanocoat formed by layer-by-layer (LBL) self-assembly confers aluminum resistance in root border cells of pea *(Pisum sativum*). J. Nanobiotechnol. 17, 53. doi: 10.1186/s12951-019-0486-y PMC6466759 30992069

[B9] He C. W. Ma J. Wang L. J. (2015). A hemicellulose-bound form of silicon with potential to improve the mechanical properties and regeneration of the cell wall of rice. New Phytologist 206, 1051–1062. doi: 10.1111/nph.2015.206.issue-3 25615017

[B10] Huang J. J. Han R. Z. Ji F. Yu Y. Wang R. Hai Z.. (2022). Glucose-6-phosphate dehydrogenase and abscisic acid mediate programmed cell death induced by aluminum toxicity in soybean root tips. J. Hazardous Materials 425, 127964. doi: 10.1016/j.jhazmat.2021.127964 34891015

[B11] Jalili S. Ehsanpour A. A. Javadirad S. M. (2022). The role of melatonin on caspase-3-like activity and expression of the genes involved in programmed cell death (PCD) induced by *in vitro* salt stress in alfalfa (*Medicago sativa* L.) roots. Botanical Stud. 63, 19. doi: 10.1186/s40529-022-00348-7 PMC9188634 35689706

[B12] Jiang C. Wang J. Leng H. N. Wang X. Liu Y. Lu H.. (2021). Transcriptional regulation and signaling of developmental programmed cell death in plants. Front. Plant Sci. 12. doi: 10.3389/fpls.2021.702928 PMC8358321 34394156

[B13] Jiang D. Xu H. Zhang Y. Chen G. (2023). Silicon mediated redox homeostasis in the root-apex transition zone of rice plays a key role in aluminum tolerance. Plant Physiol. Biochem. 201, 107871. doi: 10.1016/j.plaphy.2023.107871 37393859

[B14] Kochian L. V. Hoekenga O. A. Piñeros M. A. (2004). How do crop plants tolerate acid soils? Mechanisms of aluminum tolerance and phosphorous efficiency. Annu. Rev. Plant Biol. 55, 459–493. doi: 10.1146/annurev.arplant.55.031903.141655 15377228

[B15] Lee J. Choi J. Park J. H. Kim M. H. Hong D. Cho H.. (2014). Cytoprotective silica coating of individual mammalian cells through bioinspired silicification. Angewandte Chemie. 53, 8056. doi: 10.1002/anie.201402280 24895209

[B16] Li X. Li Y. Mai J. Tao L. Qu M. Liu J.. (2018). Boron alleviates aluminum toxicity by promoting root alkalization in transition zone via polar auxin transport. Plant Physiol. 177, 1254–1266. doi: 10.1104/pp.18.00188 29784768 PMC6053005

[B17] Liu J. Luo X. Shaff J. Liang C. Jia X. Li Z.. (2012). A promoterswap strategy between the AtALMT and AtMATE genes increased Arabidopsis aluminum resistance and improved carbon-use efficiency for aluminum resistance. Plant J. 71, 327–337. doi: 10.1111/j.1365-313X.2012.04994.x 22413742

[B18] Ma J. Cai H. M. He C. W. Zhang W. J. Wang L. J. (2015). A hemicellulose-bound form of silicon inhibits cadmium ion uptake in rice(*Oryza sativa*)cells. New Phytologist 206, 1063–1074. doi: 10.1111/nph.2015.206.issue-3 25645894

[B19] Ma J. Sheng H. C. Li X. L. Wang L. J. (2016). iTRAQ-based proteomic analysis reveals the mechanisms of silicon-mediated cadmium tolerance in rice (*Oryza sativa*) cells. Plant Physiol. Biochem. 104, 71–80. doi: 10.1016/j.plaphy.2016.03.024 27017433

[B20] Nabipour Sanjbod R. Chamani E. Pourbeyrami Hir Y. Estaji A. (2023). Investigation of the cell structure and organelles during autolytic PCD of Antirrhinum majus “Legend White” petals. Protoplasma 260, 419–435. doi: 10.1007/s00709-022-01788-5 35759085

[B21] Pang Z. H. Peng H. Lin S. Liang Y. (2023). Theory and application of a Si-based defense barrier for plants: Implications for soil-plant-atmosphere system health. Crit. Rev. Environ. Sci. Technol. 54 (9), 722–746. doi: 10.1080/10643389.2023.2267939

[B22] Rahman S. U. Han J. C. Ahmad M. Ashraf M. N. Khaliq M. A. Yousaf M.. (2024). Aluminum phytotoxicity in acidic environments: A comprehensive review of plant tolerance and adaptation strategies. Ecotoxicol. Environ. Safety 269, 115791. doi: 10.1016/j.ecoenv.2023.115791 38070417

[B23] Shavrukov Y. Hirai Y. (2015). Good and bad protons: genetic aspects of acidity stress responses in plants. J. Exp. Botany 67, 15–30. doi: 10.1093/jxb/erv437 26417020

[B24] Tabaldi L. A. Cargnelutti D. Gonçalves J. F. Pereira L. B. Castro G. Y. Maldaner J.. (2009). Oxidative stress is an early symptom triggered by aluminum in Al-sensitive potato plantlets. Chemosphere 76, 1402–1409. doi: 10.1016/j.chemosphere.2009.06.011 19570563

[B25] Tao L. Xiao X. Huang Q. Zhu H. Feng Y. Li Y.. (2023). Boron supply restores aluminum-blocked auxin transport by the modulation of PIN2 trafficking in the root apical transition zone. Plant J. 114, 176–192. doi: 10.1111/tpj.v114.1 36721978

[B26] Tariq A. Graciano C. Sardans J. Zeng F. Hughes A. C. Ahmed Z.. (2024). Plant root mechanisms and their effects on carbon and nutrient accumulation in desert ecosystems under changes in land use and climate. New Phytologist 242, 916–934. doi: 10.1111/nph.v242.3 38482544

[B27] Tyagi W. Yumnam J. S. Sen D. Sen D. Rai M. (2020). Root transcriptome reveals efficient cell signaling and energy conservation key to aluminum toxicity tolerance in acidic soil adapted rice genotype. Sci. Rep. 10, 4580. doi: 10.1038/s41598-020-61305-7 32165659 PMC7067865

[B28] Wu H. Yang F. Li H. Li Q. Zhang F. Ba Y.. (2018). Heavy metal pollution and health risk assessment of agricultural soil near a smelter in an industrial city in China. Int. J. Environ. Health Res. 30, 174–186. doi: 10.1080/09603123.2019.1584666 30810352

[B29] Xia S. S. Liu H. Cui Y. J. Yu H. P. Rao Y. C. Yan Y. P.. (2022). UDP-N-acetylglucosamine pyrophosphorylase enhances rice survival at high temperature. New Phytologist 233, 344–359. doi: 10.1111/nph.v233.1 34610140

[B30] Xiao Z. X. Ye M. J. Gao Z. X. Jiang Y. S. Zhang X. Y. Nikolic N.. (2022). Silicon reduces aluminum-induced suberization by inhibiting the uptake and transport of aluminum in rice roots and consequently promotes root growth. Plant Cell Physiol. 63, 340–352. doi: 10.1093/pcp/pcac001 34981810

[B31] Yamamoto Y. Kobayashi Y. Devi S. R. Rikiishi S. Matsumoto H. (2003). Oxidative stress triggered by aluminum in plant roots. Plant Soil 255, 239–243. doi: 10.1023/A:1026127803156

[B32] Yan L. Li S. Cheng J. Liu Y. Liu J. Jiang C. (2022). Boron contributes to excessive aluminum tolerance in trifoliate orange (*Poncirus trifoliata* (L.) Raf.) by inhibiting cell wall deposition and promoting vacuole compartmentation. J. Hazardous Materials 437, 129275. doi: 10.1016/j.jhazmat.2022.129275 35714543

[B33] Yan L. Riaz M. Li S. Cheng J. Jiang C. (2023). Harnessing the power of exogenous factors to enhance plant resistance to aluminum toxicity; a critical review. Plant Physiol. Biochem. 203, 108064. doi: 10.1016/j.plaphy.2023.108064 37783071

[B34] Yan L. Riaz M. Liu J. Liu Y. Zeng Y. Jiang C. (2021b). Boron reduces aluminum deposition in alkali-soluble pectin and cytoplasm to release aluminum toxicity. J. Hazardous Materials 401, 123388. doi: 10.1016/j.jhazmat.2020.123388 32653794

[B35] Yan L. Riaz M. Liu J. Zeng Y. Yu M. Jiang C. (2021a). The aluminum tolerance and detoxification mechanisms in plants; recent advances and prospects. Crit. Rev. Environ. Sci. Technol. 52, 1491–1527. doi: 10.1080/10643389.2020.1859306

[B36] Yang G. Qu M. Xu G. Li Y. Li X. Feng Y.. (2022). pH-Dependent mitigation of aluminum toxicity in pea (Pisum sativum) roots by boron. Plant Sci. 318, 111208. doi: 10.1016/j.plantsci.2022.111208 35351298

[B37] Ye C. J. Zheng S. Y. Jiang D. G. Lu J. Q. Huang Z. N. Liu Z. L.. (2021). Initiation and execution of programmed cell death and regulation of reactive oxygen species in plants. Int. J. Mol. Sci. 22, 12942. doi: 10.3390/ijms222312942 34884747 PMC8657872

[B38] Yu H. N. Liu P. Wang Z. Y. Chen W. R. Xu G. D. (2011). The effect of aluminum treatments on the root growth and cell ultrastructure of two soybean genotypes. Crop Protection 30, 323–328. doi: 10.1016/j.cropro.2010.11.024

[B39] Zhang J. Zou W. H. Li Y. (2015). Silica distinctively affects cell wall features and lignocellulosic saccharification with large enhancement on biomass production in rice. Plant Sci. 239, 84–91. doi: 10.1016/j.plantsci.2015.07.014 26398793

[B40] Zhu X. Wang P. Bai Z. Herde M. Ma Y. Li N.. (2022). Calmodulin-like protein CML24 interacts with CAMTA2 and WRKY46 to regulate ALMT1-dependent Al resistance in Arabidopsis thaliana. New Phytologist 233, 2471–2487. doi: 10.1111/nph.v233.6 34665465

